# Skin-on-a-chip model simulating inflammation, edema and drug-based treatment

**DOI:** 10.1038/srep37471

**Published:** 2016-11-21

**Authors:** Maierdanjiang Wufuer, GeonHui Lee, Woojune Hur, Byoungjun Jeon, Byung Jun Kim, Tae Hyun Choi, SangHoon Lee

**Affiliations:** 1 Department of Plastic and Reconstructive Surgery, Institute of Human-Environment Interface Biology, College of Medicine, Seoul Nat’l University, Seoul, Republic of Korea; 2 Biomedical Research Institute, Seoul Nat’l Univ. Hospital, Seoul, Republic of Korea; 3 KU-KIST Graduate School of Converging Science and Technology, Korea University, Republic of Korea; 4 School of Biomedical Engineering, College of Health Science, Korea University, Seoul, Republic of Korea

## Abstract

Recent advances in microfluidic cell cultures enable the construction of *in vitro* human skin models that can be used for drug toxicity testing, disease study. However, current *in vitro* skin model have limitations to emulate real human skin due to the simplicity of model. In this paper, we describe the development of ‘skin-on-a-chip’ to mimic the structures and functional responses of the human skin. The proposed model consists of 3 layers, on which epidermal, dermal and endothelial components originated from human, were cultured. The microfluidic device was designed for co-culture of human skin cells and each layer was separated by using porous membranes to allow interlayer communication. Skin inflammation and edema were induced by applying tumor necrosis factor alpha on dermal layer to demonstrate the functionality of the system. The expression levels of proinflammatory cytokines were analyzed to illustrate the feasibility. In addition, we evaluated the efficacy of therapeutic drug testing model using our skin chip. The function of skin barrier was evaluated by staining tight junctions and measuring a permeability of endothelium. Our results suggest that the skin-on-a-chip model can potentially be used for constructing *in vitro* skin disease models or for testing the toxicity of cosmetics or drugs.

The main function of human skin is to protect organs by serving as a physiological barrier and such, skin is exposed to many chemical substances and biological agents, including cosmetics, skin detergents, ultraviolet light, pathogens, environmental pollutants and micro-organisms. Rapid increases in these factors can cause various skin reactions, such as skin inflammation, irritation, allergies and even cancer; thus, a substantial need to screen the toxicity of certain materials and the efficacy of drugs for the skin has arisen. For this purpose, several millions of animal experiments, mainly in mice, have been performed all over the world^1^,^2^; however, animal studies have two critical limitations. The first comprises ethical and regulatory issues, and the second is the considerable difference between mouse and human skin, i.e., in thickness, hair density, and appendages^2^,^3^. Moreover, with the exception of the footpads, mouse skin does not have sweat glands. According to Humane Society International, 9 out of 10 candidate medicines that appear safe and effective in animal studies fail when administered to humans, and animal studies often fail to predict actual human outcomes less than 10 percent of cases^4^,^5^. Due to these reasons, there is an urgent need to establish surrogate *in vitro* systems that mimic human skin as closely as possible. Since the first report of human skin-like constructs in the early 1980s^6^, diverse *in vitro* skin models have been developed and commercialized^7^; however, most of these models are based on fibroblasts and keratinocytes and employ static culture systems that only emulate human epidermis. The complicated structure of the skin cannot be mimicked by these cells alone because the skin contains many hair follicles, immune cells, melanocytes, Merkel cell complexes, blood vessels, nerve fibers and multilayered structures. Therefore, researchers in a wide variety of industrial, clinical and academic fields are anticipating the development of *in vitro* skin models capable of simulating critical and common skin diseases.

Among the skin diseases, a number of people suffer from inflammatory skin disease. Inflammation is a common physiological and pathological response that occurs to protect a host from infection with foreign organisms. Inflammation can also occur in response to physical stimuli, and acute inflammation is the initial protective response to external stimuli. In this process, the movement of body fluids, including plasma and leukocytes, from the blood into the locally stimulated tissue increases, causing edema. This inflammatory response in injured tissue initiates the innate immune system in the skin, activating cells, such as macrophages, epidermal dendritic cells and Langerhans cells. The host reactions to external stimuli cause the release of inflammatory mediators, including proinflammatory cytokines and chemokines such as interleukin-1 beta (IL-1β), IL-6, IL-8 and tumor necrosis factor-α (TNF-α)^8^,^9^. Previous experiments have shown that the expression of inflammatory mediators is increased in inflammatory skin lesions^10^,^11^,^12^. The proinflammatory factors IL-1β, IL-6, IL-8 and TNF-α play a key role in the initial phase of inflammation^13^,^14^,^15^,^16^,^17^. Although tissue engineered skin and *in vitro* human skin models have been developed for a variety of applications, such as constructing skin-related disease models and assessing the penetration of chemicals or transdermal drugs during the past three decades^7^,^18^,^19^,^20^,^21^,^22^,^23^, it has been one of challenges to mimic skin disease model including inflammation and edema. Most of the current commercially available human skin models consist of integrated epidermal keratinocytes and dermal fibroblasts used for pharmaceutical testing in the epidermal or dermal compartment alone or in both^7^,^20^,^23^,^24^,^25^,^26^. Furthermore, recent advances in microfluidic systems have enabled the modeling of the physiology of various organs. These so-called organ-on-a-chip systems, including skin-on-a-chip devices^19^,^27^,^28^, have the potential to offer physiologically relevant disease modeling and drug evaluation. Despite the considerable effort that has been directed toward constructing an *in vitro* model of skin, especially for edema evaluation, challenges remain because there are no skin-on-a-chip devices fully consisting of epidermal, dermal and endothelial layers.

In this study, we used microfluidic technology to develop a human skin-on-a-chip model consisting of three layers, i.e., epidermal, dermal and vascular layer. The microfluidic device is designed for dynamic cell culture and was constructed with 3 layers and each layer is separated using transparent, porous membranes to allow interlayer communication and mimic skin biology. The chip is designed to culture large number of cells and post structures were employed to maintain the gap between membranes. TNF-α was perfused through the device to induce inflammation and edema in the skin-on-a-chip model, and proinflammatory cytokine and chemokine levels were then analyzed. Furthermore, we used dexamethasone (Dex) to evaluate the efficacy as drug testing model. We stained tight junctions to evaluate a TNF-α-induced inflammatory edema response and subsequent drug effects in human umbilical vein endothelial cells (HUVECs). We expect that the proposed model could also be used for simulating other critical inflammatory skin diseases, such as eczema and for testing the toxicity of cosmetics or drugs.

## Results

### Cell culture in human skin-on-a-chip device

**Figure 1:**
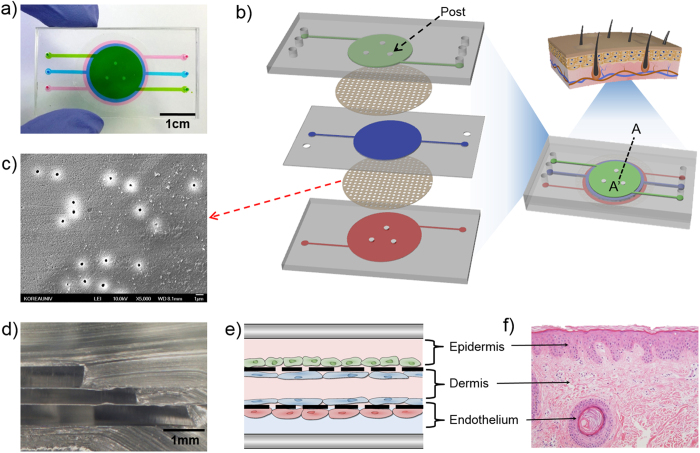
Description of the microfluidic device. (**a**) Image of a skin-on-a-chip device filled with fluid three different colors. (**b**) 3D scheme of the skin-on-a-chip system comprising three PDMS layers and two PET porous membranes (footprint: 48 mm × 26 mm; height: 7 mm). (**c**) SEM image of PET porous membranes (pore size: 0.4 μm) obtained from Transwell. (**d**) Cross-sectional image of A–A’. (**e**) Schematic of the skin-on-a-chip system, including three separate channels with four vertically stacked cell layers. (**f**) Representative histological skin section stained with hematoxylin and eosin to indicate the cellular organization of skin.

**Figure 2:**
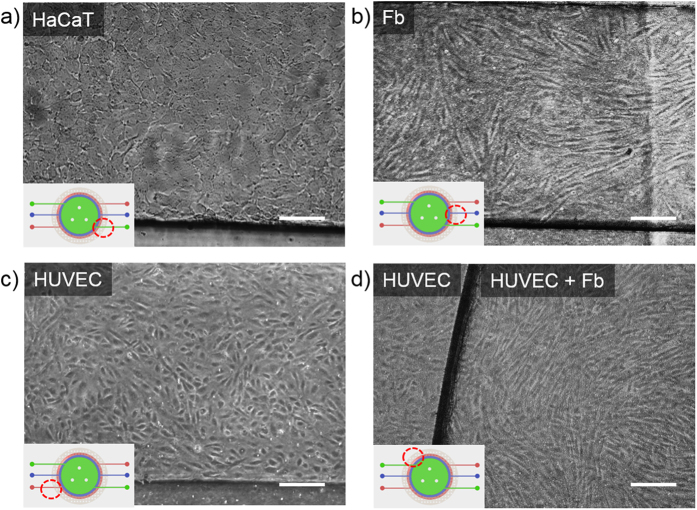
Optical images of cells in a skin-on-a-chip device. Images of confluent HaCaT (**a**), Fb (**b**) and HUVEC (**c**) monolayers at day 3 in the microfluidic devices. (**d**) Fbs and HUVECs were cultured on the top and bottom, respectively, of the lower porous membrane in the microfluidic device. The red-dotted circle in each figure depicts the location of the area shown in the microscopic image. Scale bars: 150 μm. HaCaT, human immortalized keratinocyte; Fb, fibroblast; HUVEC, human umbilical vein endothelial cell.

The stability of the proposed skin chip was tested. The APTES-based bonding between the PET membranes and PDMS channels enabled up to 3 weeks of cell culture without leakage. Each chamber was designed to contain a sufficient number of cells for performing gene expression analyses using conventional methods; the volume of the top, middle and bottom chamber is approximately 110 μl, 90 μl and 173 μl, respectively. HaCaTs and Fbs were seeded onto the upper layer and the middle layer, respectively, and the cells were incubated overnight for the cells to adhere to the membrane. Then, the chip was turned over, and Fbs and HUVECs were seeded on the middle layer and the bottom layer, respectively. The three types of cells were cultured until reaching confluence in order to mimic the epithelial and endothelial barriers. Figure 1d shows a cross-sectional view of the edge of the cell culture chambers. As shown in Fig. 2, all cell types were confluent over the membranes by day 3 (Fig. 2). HaCaTs (Fig. 2a), Fbs (Fig. 2b) and HUVECs (Fig. 2c) were cultured on the porous membranes in the microfluidic chip. The different size of the three chambers allows the cells to be observed at the edge of each cell chamber during culture.

**Figure 3:**
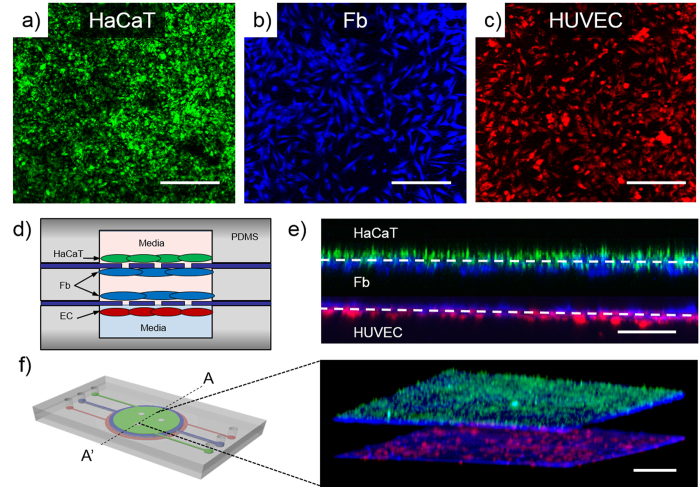
Images of (**a**) HaCaTs, (**b**) Fbs and (**c**) HUVECs immunostained with Cell Tracker^TM^. (**d**) Schematic of the side view of a skin-on-a-chip device. (**e**) Four layers with three cell types were stacked on two porous membranes. Z-stacked fluorescence image showing that all cells adhered to the PET membranes. (**f**) 3D fluorescence image of the cross section (A-A’) of a microfluidic device. Scale bars: 300 μm.

To investigate the proper formation of the skin-mimicking layers, we labeled each cell type using Cell Tracker^TM^. HaCaTs, Fbs and HUVECs were stained with Cell Tracker^TM^ Green CMFDA, Blue and Red CMPTX, respectively, and the results are shown in Fig. 3a–c. Figure 3d and e show a schematic side view and an image of immunostained cells in the skin chip. Cells were successfully grown on the front and back sides of the membranes, indicating that the proposed cell seeding and culturing method is feasible. 3D fluorescence images (Fig. 3f) of stacked cells in the skin chip (Fig. 3e) demonstrated that all types of cells were cultured uniformly over the large area of the membranes, forming a layered structure.

### Inflammation induction and evaluation

**Figure 4:**
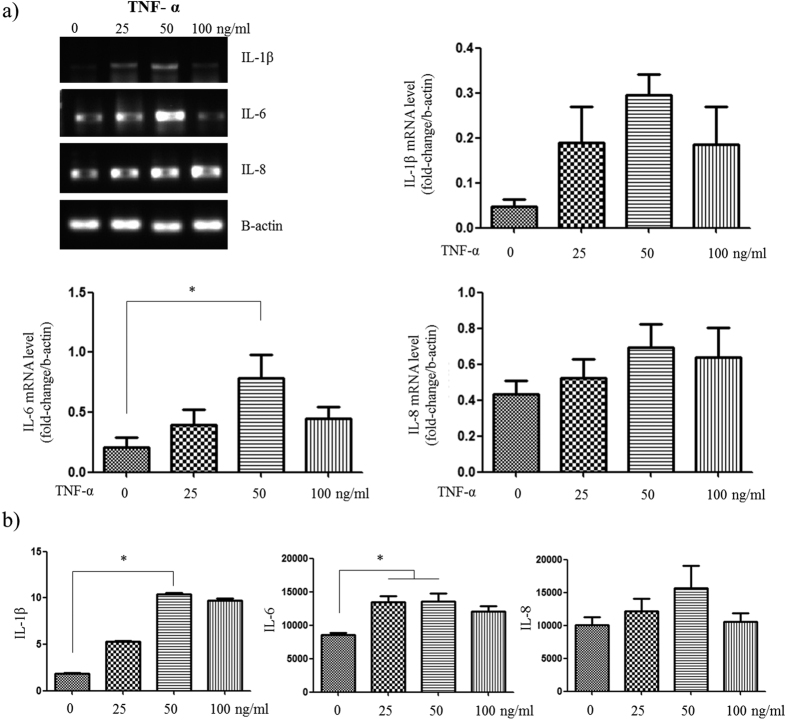
Inducing inflammation in the skin-on-a-chip system. (**a**) Representative figures showing the mRNA expression levels and intensity for the pro-inflammatory mediators (IL-1β, IL-6 and IL-8) in HUVECs stimulated by TNF-α. (**b**) Levels of IL-1β, IL-6 and IL-8 secreted from HUVECs following stimulation with different concentrations of TNF-α for 24 hours, as measured by a multiplex assay (n = 3, *p < 0.05).

To optimize the concentration of perfused TNF-α for inducing inflammation in the skin-mimicking model, the Fb layer was treated with various concentrations of TNF-α (0, 25, 50, and 100 ng/ml) for 24 hours. After 24 hours of incubation, the HUVECs and medium in the bottom layer were harvested, and proinflammatory cytokine, chemokine and mRNA expression levels were quantified by RT-PCR (Fig. 4a). IL-1β and IL-6 mRNA were strongly expressed after treatment with 50 ng/ml TNF-α. The level of IL-8 mRNA expression increased according to TNF-α in a dose-dependent manner.

The medium in the bottom layer was analyzed using ELISA (Fig. 4b). The data showed that IL-1β, IL-6 and IL-8 levels were increased in a dose-dependent manner with concentrations ranging from 0, 25, and 50 ng/ml but were decreased after treatment with 100 ng/ml. In particular, compared with no TNF-α treatment, the 50 ng/ml TNF-α treatment led to significantly increased IL-1β and IL-6 levels (*p < 0.05). Therefore, 50 ng/ml was determined to be the most appropriate concentration of TNF-α for inducing experimental inflammation in the skin-on-a-chip system. Based on this result, we adopted the skin chip exposed to 50 ng/ml TNF-α as an inflammation model for further study.

### Drug treatment on skin chip

**Figure 5:**
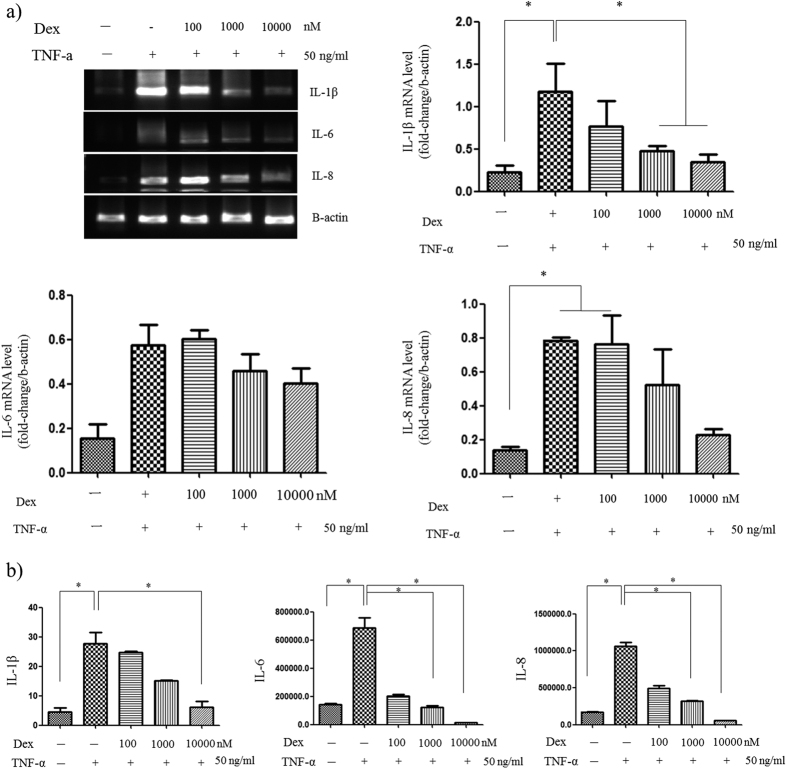
Pharmaceutical treatment of the skin-on-a-chip system. (**a**) The levels and intensity of secreted pro-inflammatory cytokine mRNA in HUVECs were analyzed by RT-PCR. (**b**) The released levels of IL-1β1b, IL-6, and IL-8 in the HUVEC culture medium were measured by a multiplex assay (n = 3, *p < 0.05).

Dex is a popularly used drug for treating TNF-α-induced inflammation. To simulate the process by which Dex treats inflammation, various doses of Dex were applied to the HaCaT layer, and we pretreated the HaCaT layer that was damaged by 50 ng/ml TNF-α. Subsequently, the mRNA levels of the proinflammatory markers IL-1β, IL-6, and IL-8 were measured; the results are shown in Fig. 5b. As the dose of Dex increased from 100 nM to 10,000 nM, the expression levels of IL-1β, IL-6, and IL-8 decreased, indicating alleviation of the inflammation (Fig. 5a).

To further investigate the effect of Dex, we used ELISA to analyze the medium in the bottom chamber (Fig. 5b). The results indicated that the IL-1β, IL-6, and IL-8 protein levels all significantly increased after the TNF-α 50 ng/ml treatment; in addition, all these levels decreased with increasing Dex pretreatment doses (from 100 nM to 10,000 nM). Pretreatment with 1,000 nM Dex led to decreased IL-6 and IL-8 protein level after treatment with 50 ng/ml TNF-α compared to no pretreatment. Moreover, pretreatment with 10,000 nM Dex further decreased the IL-1β, IL-6, and IL-8 protein levels compared to no pretreatment after treatment with 50 ng/ml TNF-α, suggesting that the low values were induced by the high Dex dose. As a result, the most appropriate concentration of Dex for the drug treatment experiment was determined to be 1,000 nM.

### Protective effects of Dex against TNF-α-induced tight junction structure disruption

**Figure 6:**
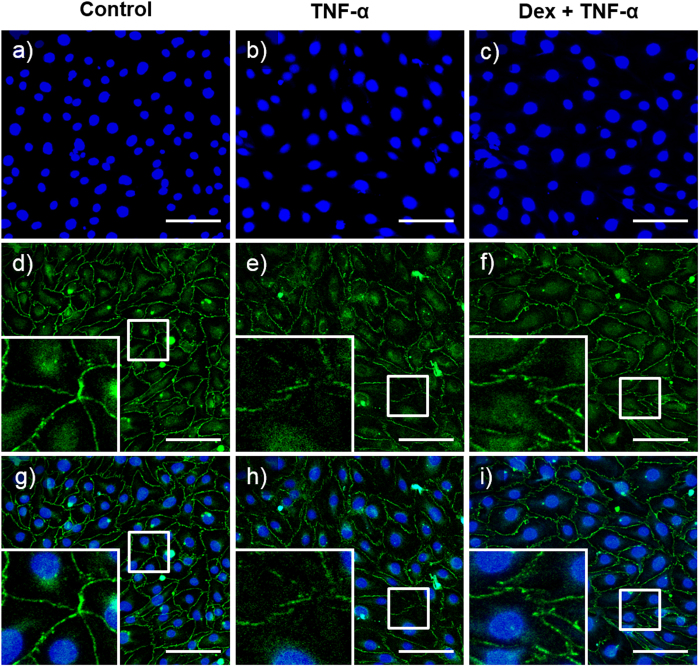
Immunocytochemical tight junction staining in HUVECs. Distribution of the tight junction protein ZO-1 in a non-treated chip (control), a TNF-α-treated (50 ng/ml) chip and a Dex-treated (1,000 nM) chip, as observed with immunofluorescence microscopy. (**a–c**) DAPI staining showing the distribution of HUVEC nuclei. (**d–f**) Images of ZO-1 staining and (**g–i**) merged images depicting the HUVEC tight junctions. Gaps were observed in the TNF-α-treat chip (**e,h**), and Dex prevented this TNF-α-induced disruption of the tight junctions (**f,i**). Scale bars: 100 μm. Dex, Dexamethasone.

The paracellular permeability of endothelial cells is controlled by protein complexes, such as occludins, claudins and zonula occludens (ZO), which act as a fluid permeability barrier. To evaluate the effect of the TNF-α and Dex treatments on tight junction formation, we performed immunocytochemical staining of the tight junction protein ZO-1 (Fig. 6). Based on the previous results, we used 1,000 nM Dex for the paracellular permeability assay. HUVECs in the bottom layer of an unstimulated chip (control), a TNF-α-treated chip and a Dex-treated chip were stained using DAPI and a marker for ZO-1. Figure 6(a–c) shows the distribution of HUVEC nuclei in each chip group. The ZO-1 distribution patterns were observed with immunofluorescence microscopy. The staining for the tight junction protein ZO-1 in the control was observed to be linear and localized compared to that in the test conditions (Fig. 6d). In contrast, the distribution of ZO-1 in the chip treated with TNF-α for 24 hours (Fig. 6e) was not circumferentially continuous and was more punctuate. High-magnification immunofluorescence confocal imaging showed that TNF-α inhibited the formation of tight junctions. This TNF-α-induced inhibition was reduced by Dex. The relative intensities of immunocytochemical tight junction staining in HUVECs were measured to quantify the fluorescence of tight junction (Supplementary Fig. 2). We set the intensity of control group to 100% to direct compare. The intensity of TNF-α treat group was about 38% and the intensity of Dex-treate group was about 82%. We confirmed the protective effect of Dex on tight junctions exposed to TNF-α; the upper layer was treated with Dex for 24 hours before the middle layer was treated with TNF-α. As shown in Fig. 5f, exposing the Dex-treated chip to TNF-α had no considerable effect on ZO-1 abundance.

### Modeling skin edema in the skin-on-a-chip device

**Figure 7:**
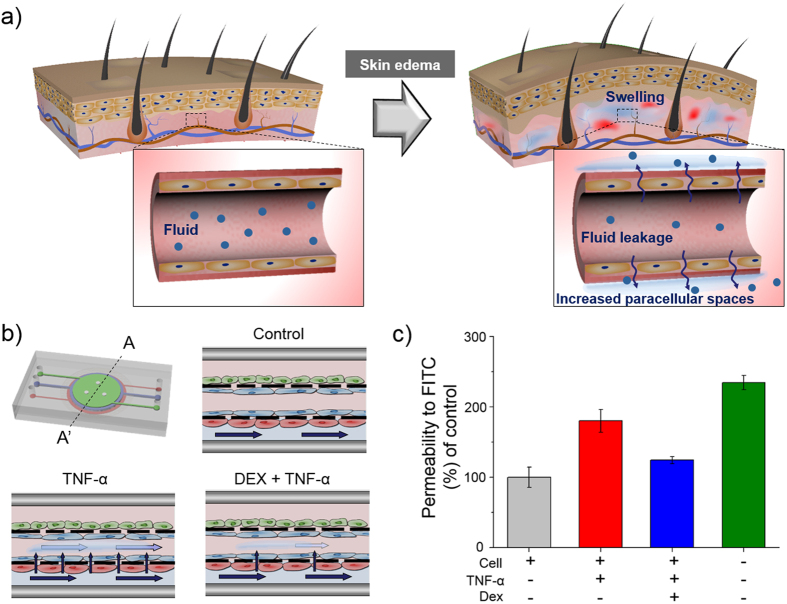
Permeability and therapeutic analysis of the on-chip skin edema model. (**a**) Schematic of the human skin edema model. Inflammation induced by TNF-α damages tight junctions, resulting in vascular leakage. (**b**) Schematic of the skin edema model in a microfluidic device with TNF-α exposure after a Dex pretreatment (**c**) TNF-α-treated chips exhibited increased paracellular permeability to FITC-dextran (4 kDa) compared with non-treated chips. The permeability of Dex-treated chips was significantly lower than that of chips treated only with TNF-α.

Edema is swelling resulting from fluid accumulating in the interstitium from the vasculature when inflammation occurs in the body (Fig. 7a). Thus, we explored whether skin edema could be mimicked *in vitro* in the skin-on-a-chip device. Pathological changes in fluid transport were quantitatively examined by measuring the paracellular permeability of the engineered vascular and dermal layers to FITC-dextran (4 kDa). FITC-dextran was injected in the engineered vascular layer in the three chip types, i.e., an unstimulated chip (control), a TNF-α-treated chip and a Dex-treated chip. The level of FITC-dextran transported from the vascular layer (bottom layer) to the dermal layer (middle layer) was measured by collecting the fluid of the middle layer after 1 hour of incubation (Fig. 7b). The permeability of the TNF-α-treated chip was 1.8 times higher than that of the control chip. The chip treated with both Dex and TNF-α exhibited paracellular permeability 1.2 times greater than that of the control chip (Fig. 7c).

## Discussion

In the present study, we successfully constructed an *in vitro* human skin-on-a-chip device to simulate skin inflammation and edema. Recently, several groups have reported work on skin-on-a-chip systems^19^,^27^,^29^, and most of such skin models have been established by co-culturing dermis, epidermis or immune cells with skin cells. To the best of our knowledge, this is the first study to show a vascularized skin model with inflammation and edema achieved by co-culturing endothelium with skin cells. We developed a microfluidic device consisting of three layers to mimic the epidermis, dermis and vessels in the skin, and diverse culture media can be provided to each skin layer with different flow rates. We used PET porous membranes between each skin layer to allow macromolecules, such as cytokines, nutrients and drugs, to diffuse into other skin layers. We used HaCaT cells, HS27 fibroblasts (Fbs), and HUVECs as substitutes for the epithelial cells, Fbs and blood vessel endothelial cells present in skin tissue. HUVEC is commonly used for endothelial cell related research, so we can compare our result with preceding research. Fibronectin coating was applied to each membrane to mimic the extracellular matrix (ECM)^30^. The proposed model successfully simulated skin inflammation and edema. We investigated TNF-α-induced skin inflammation by analyzing proinflammatory cytokine (IL-1β, IL-6) and chemokine (IL-8) levels^31^. Moreover, the model demonstrated the prevention of inflammation by the application of a drug. The tight junction damage was prevented by the application of Dex to the skin edema model, and the expression of IL-1β, IL-6, and IL-8 was decreased, indicating the recovery of skin with edema. These results showed the strong potential application of *in vitro* skin-on-a-chip models for screening drugs and replacing animal experiments. One of the crucial challenges of fabricating these microfluidic chips is irreversibly bonding the PDMS and porous PET membrane to prevent fluid leakage. In this study, the membrane and PDMS were irreversibly bonded by silanization with APTES^32^,^33^. The chip size was optimized for further molecular assays, and posts were designed to maintain the gap between membranes. These posts play a crucial role in increasing the cell culture area in the chip. Without them, the large membranes bent downward, and the chambers collapsed. Using optical microscopy to observe cultured cells in multiple layers of microfluidic channels of equal size and shape is challenging because the cells and membranes are overlapped. To address this problem, the area of each chamber was designed to be different, thereby allowing cells to be observed with optical microscopy.

Inflammation is an important factor in skin pathologies. To induce skin inflammation on a chip, we studied a well-known TNF-α and NF-kB signaling pathway (Supplementary Fig. 1)^34^,^35^,^36^,^37^. In brief, TNF-α activates NF-kB and increases the degradation of IkB^38^. Dex increases the level of IkB, and NF-kB remains bound to IkB. TNF-α has been shown to promote the immune/inflammatory activation and induction of IL-1β, IL-6, and IL-8, and play a key role in various immunological disorders and inflammation in the skin^39^,^40^. Moreover, TNF-α induces alterations in endothelial cytoskeletal actin and the formation of intercellular gaps, causing increased permeability to macromolecules^40^,^41^. As such, we applied TNF-α to induce inflammation, and the experimental results showed that the proposed model reflects this pathological mechanism.

After establishing the inflammation model in the chip, we considered the use of transdermal drug treatments in the inflammation model. Skin inflammation is routinely treated with steroids, and Dex is commonly used to inhibit TNF-α induced inflammation^34^,^42^,^43^,^44^,^45^. When Dex was applied to the skin damaged by TNF-α, the release of IL-1β, IL-6 and IL-8 was decreased depending on the Dex pretreatment dose; this result is similar to those of previously reported work^46^,^47^. To the best of our knowledge, this is the first *in vitro* skin model to simulate the recovery of a pathological skin condition by a drug.

Another feature of the proposed skin model is that skin edema, which is the most challenging *in vitro* skin model, was established. Skin irritation is the result of chemicals penetrating through the stratum corneum and damaging keratinocytes or other skin cells. The increased permeability of endothelial cells produces erythema and edema^48^. Currently, reconstructed human epidermis (RhE)-based test methods do not include any vascularization and only measure cell/tissue damage by testing cell viability^49^,^50^. The damaged cells release inflammatory cytokines and induce an inflammatory cascade, which also acts on the dermis and the endothelial cells of the blood vessels; it is not possible to detect these effects using current RhE models. Our skin-on-a-chip model consists of epidermal, dermal and endothelial cells, which can be used to simulate skin edema. Edema is caused by excessive capillary permeability, and paracellular permeability is effected by complexes called tight junctions^51^,^52^. Therefore, we observed state of tight junctions with immunostaining, and our results suggest not only that tight junction loss occurred under proinflammatory conditions but also that this tight junction loss could be prevented by Dex (Fig. 6). We also observed and quantitatively analyzed fluid leakage from the endothelium to the dermis using FITC-dextran. This study demonstrated that Dex attenuates endothelial barrier dysfunction stimulated by TNF-α in this *in vitro* skin-on-a-chip system. Our experiment suggests that this system could potentially be used to replace related animal experiments and to develop new drugs and cosmetics.

## Conclusion

In this study, we constructed a miniature model of human skin in a microfluidic platform consisting of epidermal, dermal and endothelial layers. We optimized the concentration of TNF-α to induce inflammation and edema and the inflammation was observed by analyzing the expression levels of the proinflammatory factors IL-1β, IL-6 and IL-8. Furthermore, we demonstrated that Dex reduced the TNF-α-induced inflammation and edema, in accordance with our data of tight junction staining and permeability testing data. The tested drug, i.e., Dex, diffused from the epidermal layer to the endothelial layer through the porous membrane and protected the endothelial layer from inflammation and edema. These experiments simulated the common application of ointments to epithelium to treat inflammation and edema. Thus, our findings suggest that this *in vitro* microfluidic skin chip can mimic the structure and physiological function of human skin by co-culturing epidermal, dermal and endothelial cells. We expect that the proposed approach could potentially be used in clinical application, as well as in the cosmetic and pharmaceutical industries.

## Materials and Methods

### Design and fabrication of human skin-on-a-chip system

The microfluidic device for simulating skin was fabricated using conventional soft lithography with an elastomeric material, polydimethylsiloxane (PDMS)^53^ (Fig. 1a). The upper, middle and lower layers of the microfluidic chip were prepared by casting PDMS prepolymer on the master mold fabricated using photoresist (SU8–100, MicroChem). The diameter of each chamber was designed to be different in size, allowing cells to be observed using an inverted microscope. The upper, middle and lower layer chambers were 16 mm × 550 μm, 18 mm × 350 μm, and 20 mm × 550 μm (diameter × height) in size, respectively. Porous membranes were placed between each layer to separate the chambers. The porous membranes were obtained from Transwell (24 mm diameter, 0.4 μm pore size, Corning), and bonding between the PDMS and the polyester (PET) membranes was achieved by amino-silanizing the PET membranes^32^. For the amino-silanization, the PET membrane was treated with oxygen plasma for 30 seconds (600 mTorr, 80 W) and then immersed for 30 minutes in a 5% solution of 3-aminopropyltriethoxysilane (APTES, Sigma-Aldrich) diluted in water that had been pre-warmed with a hot plate to 80 °C. Then, the PET membrane was washed with deionized water and dried at room temperature. The amino-silanized PET membrane and PDMS microchip were then treated with oxygen plasma for 30 seconds (600 mTorr, 80 W) and bonded. We used microscope, methanol and tweezer in all alignment process. Finally, the skin chip consisting of three PDMS layers and two PET membranes was baked in an oven for 24 hours at 60 °C (Fig. 1b).

### Cell culture

We used HaCaT cells, HS27 fibroblasts (Fbs), and HUVECs as substitutes for the epithelial cells, Fbs and blood vessel endothelial cells present in skin tissue^54^. The human epidermal keratinocytes (HaCaT, ATCC^®^, USA) and human skin Fb HS27 cells (CRL-1634, ATCC^®^, USA) were cultured in high-glucose DMEM (WELGENE, South Korea) supplemented with 10% fetal bovine serum (FBS, Gibco, Grand Island, NY) and 1% penicillin/streptomycin solution (Gibco, Grand Island, NY). The HUVECs (PCS-100-010^™^, ATCC^®^, USA) were cultured in EGM-2 supplemented with ascorbic acid, vascular endothelial growth factor, 20% FBS, recombinant human Fb growth factor, hydrocortisone, insulin-like growth factor-1, a recombinant analog of human insulin-like growth factor-1, gentamicin–amphotericin, and heparin (Lonza, Walkersville, MD, USA). All experiments used cells from passages 3–10.

### Preparation of skin-on-a-chip system

The microfluidic device was first sterilized by ethylene oxide gas, and the PET membranes were coated with fibronectin (20 μg/ml) for 24 hours in 4 °C, rinsed with PBS and then dried for 24 hours in a sterile hood at room temperature. Fbs and HUVECs were both seeded at a density of 1 ×10^6^ cells/ml in the middle and bottom layers. Then, the microfluidic device was turned over to allow cell adhesion to the porous membrane and incubated at 37 °C. After 4 hours of cell attachment, the microfluidic device was overturned, and Fbs and HaCaT cells were both seeded at a density of 1 × 10^6^ cells/ml in the middle and top layers and incubated at 37 °C. The culture medium was changed every day by gravity driven flow, and all types of cells in all three layers reached confluence within 3 days.

### Cell labeling

Cell Tracker^TM^ fluorescent probes were used to demonstrate the three-dimensional positions of all three cell types. In this study, Cell Tracker^TM^ Red CMPTX (C34552), Blue (C12881), and Green CMFDA (C7025) (Molecular Probes, Invitrogen) were used to label cells. After the cells were cultured in the appropriate medium, the medium was removed, and then pre-warmed (37 °C) Cell Tracker^TM^ working solution (0.1 μM) that had been prepared in serum-free medium was gently added; the cells were incubated with this solution for 45 minutes at 37 °C. Then, the cells were trypsinized, centrifuged and seeded into the microfluidic device. After the cells in all three layers reached confluence, they were imaged under a fluorescence microscope.

### Measurement of paracellular permeability

Fluorescein isothiocyanate (FITC)-dextran (Sigma, St. Louis, MO, USA) movement across the HUVEC and Fb monolayers was used to examine the paracellular permeability of the endothelial barrier. In brief, at the end of an experiment, the middle layers of three individual chips, including a non-treated chip (control), a TNF-α (Sigma, St. Louis, MO, USA)-treated chip and a Dex (Sigma, St. Louis, MO, USA)-treated chip, were gently washed and placed in HBSS. Medium in the bottom layer was aspirated and replaced with 200 μl of 1 mg/ml FITC-dextran dissolved in HBSS. After the chips were incubated at 37 °C for 24 h, 100 μl samples were obtained from the middle layer of each chip, and the fluorescence intensity of the fluid was measured at excitation and emission wavelengths of 485 nm and 538 nm, respectively, using a microplate reader (Molecular Devices, Sunnyvale, CA).

### Immunofluorescence imaging

The three different cell types in the layers were fixed by 4% paraformaldehyde (PFA) for 30 minutes at 4 °C and then rinsed with PBS containing 0.1% BSA. Subsequently, 0.1% Triton X-100 in PBS was introduced to the chip layers and allowed to stand for 15 minutes at room temperature. After being washed with PBS containing 0.1% BSA, the chip was immersed in 3% BSA at room temperature for 30 minutes. After another wash with PBS containing 0.1% BSA, the chip was incubated overnight at 4 °C with anti-ZO-1 tight junction protein antibody (ZO1-1A12, Invitrogen). The chip was washed with 0.1% BSA again and incubated at room temperature for 60 minutes with Alexa Fluor 488-conjugated anti-mouse IgG (Invitrogen) secondary antibodies. Then, the chip was incubated with DAPI (4′,6-diamidino-2-phenylindole) for 10 minutes at room temperature. After the chip was washed with PBS once more, immunofluorescence images were obtained using a confocal microscope (Olympus, Japan).

### RT-PCR

Total RNA was isolated using an RNeasy mini kit (Qiagen, USA) according to the manufacturer’s instructions to examine the mRNA expression levels of IL-1β, IL-6 and IL-8 after the drug treatment. Total RNA concentrations were determined using a UV-Vis spectrophotometer and absorbance at 260 nm (NanoDrop 2000, Thermo Scientific, USA). For RT-PCR, 1 μg of mRNA was reverse transcribed with oligo (dT) 18 using a thermal cycler (C-1000 Touch, Bio-Rad, USA). PCR was performed using mixtures containing Taq DNA polymerase (Thermo Scientific, USA) and the appropriate primers (Table 1) with a thermal cycler (C-1000 Touch, Bio-Rad, USA) and the following parameters: 95 °C for 5 minutes (I), 95 °C for 30 seconds (II), 53–58 °C for 30 seconds (III), 72 °C for 1 minute (IV) followed by 40 cycles (II-IV), 72 °C for 7 minutes (V), and 4 °C for overnight (VI).

### Multiplex assay

A multiplex immunoassay analysis was performed to measure the concentrations of multiple cytokines IL-1β set (cat #171B5001M), IL-6 set (cat #171B5006M), and chemokines IL-8 set (cat #171B5008M) in human biological fluid by using a Bio-Plex^®^ multiplex system (Bio-Rad Laboratories, Hercules, CA) with Luminex-bead-based xMAP technology according to the manufacturer’s instructions. The antibodies specific to each factor were covalently coupled to the microspheres, each of which was uniquely labeled with a fluorescent dye. The microspheres were incubated with standards and samples in a 96-well microtiter plate at 850 rpm for 30 minutes at room temperature. After washing with wash buffer, diluted biotinylated secondary antibody was added to the appropriate wells and incubated at 850 rpm for 30 minutes. After washing, phycoerythrin-conjugated streptavidin (PE-streptavidin) was added to each well and incubated for 10 minutes. A final wash removed any unbound PE-streptavidin, and the microspheres were resuspended in buffer and analyzed using the Bio-Plex^®^ multiplex system to determine the concentration of each biomarker. All of biological fluid samples were diluted a minimum 4-fold.

### Induction of inflammation

Various doses (0, 25, 50, 100 ng/ml) of TNF-α (Abcam, USA) were perfused through the Fb layer to develop an on-chip model of human skin inflammation. After the TNF-α perfusion, the chip was incubated at 37 °C for 24 hours. Then, the HUVECs and Fbs of the lower layer were harvested, and the proinflammatory cytokine (IL-1β, IL-6) and chemokine (IL-8) concentrations were measured by RT-PCR and multiplex analysis.

### Treatment of inflammation using Dex and analyses of its effect and mechanism

The HaCaT layer in the chip was pretreated with various doses of Dex (0, 100, 1,000, and 10,000 nM) for 24 hours, and then the Fb layer was exposed to TNF-α for 24 hours, leading to abnormal cytokine and chemokine expression. Then, the gene expression of TNF-α-induced proinflammatory mediators (IL-1β, IL-6, and IL-8) was analyzed by quantitative RT-PCR and ELISA.

## Additional Information

**How to cite this article**: Wufuer, M. *et al*. Skin-on-a-chip model simulating inflammation, edema and drug-based treatment. *Sci. Rep.* **6**, 37471; doi: 10.1038/srep37471 (2016).

**Publisher’s note:** Springer Nature remains neutral with regard to jurisdictional claims in published maps and institutional affiliations.

## Supplementary Material

Supplementary Information

## Figures and Tables

**Table 1:** Primers used for RT- PCR.

No.	Gene name	Sequence (5′-3′)	Primer size
1	IL-1β	Forward: GGATATGGAGCAACAAGTGG	20
		Reverse: ATGTACCAGTTGGGGAACTG	20
2	IL-6	Forward: GCCTTCGGTCCAGTTGCCTT	20
		Reverse: AGTGCCTCTTTGCTGCTTTCAC	22
3	IL-8	Forward: AGGGCCAAGAGAATATCCGA	20
		Reverse: GGATCCGGCTAGCAGACTA	19
4	β-actin	Forward: TGGCACCCAGCACAATGAA	19
		Reverse: CTAAGTCATAGTCCGCCTAGAAGCA	25
