# Supplementary material for: Skin-on-a-chip model simulating inflammation, edema and drug-based treatment
Source: Sci Rep. 2016 Nov 21;6:37471. doi: 10.1038/srep37471 (PMC5116589; doi:10.1038/srep37471)

Supporting information

Title:

Skin-on-a-chip model simulating inflammation, edema and drug-based treatment

Author list:

Maierdanjiang Wufuer[1,2†], GeonHui Lee[3†], Woojune Hur[1,2], Byoungjun Jeon[1,2], Byung Jun Kim[1], Tae Hyun Choi[1*] and SangHoon Lee[3,4*]

[1] Department of Plastic and Reconstructive Surgery, Institute of Human-Environment Interface Biology, College of Medicine, Seoul Nat'l University

[2] Biomedical Research Institute, Seoul Nat'l Univ. Hospital

[3] KU-KIST Graduate School of Converging Science and Technology, Korea University, Republic of Korea

[4] School of Biomedical Engineering, College of Health Science, Korea University, Seoul, Republic of Korea

*Corresponding Authors. E-mail: dbiomed@korea.ac.kr or psthchoi@snu.ac.kr

† These authors contributed equally as first author to this work.

Supplementary Fig. S1.

Schematic representation of TNF-α inflammation and Dex pathways.

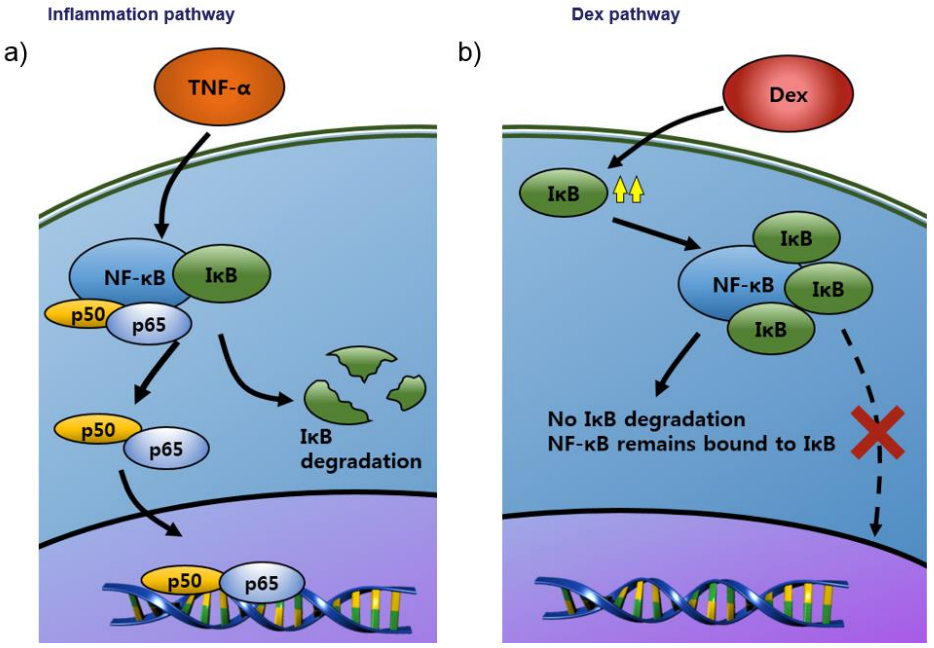

Supplementary Fig. S2.

Relative intensity comparison of immunocytochemical tight junction staining in HUVECs

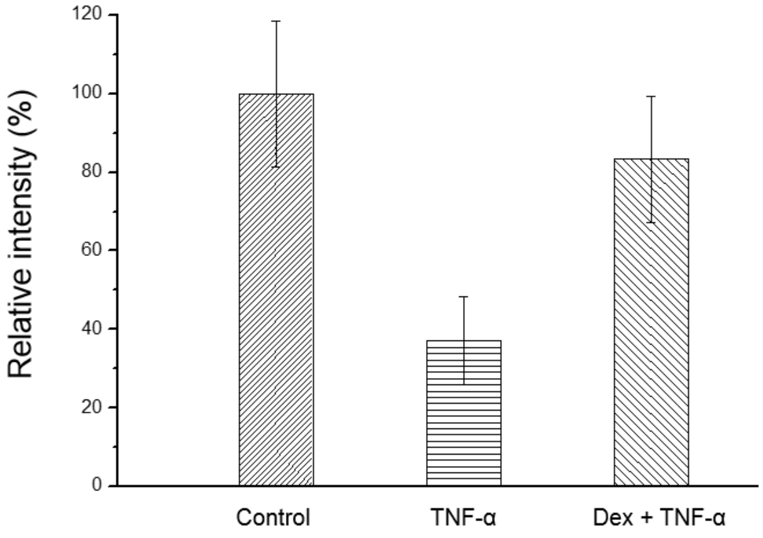

Supplement: Supplementary Information [file srep37471-s1.pdf]
